# Simulation of crop growth, time to maturity and yield by an improved sigmoidal model

**DOI:** 10.1038/s41598-018-24705-4

**Published:** 2018-05-04

**Authors:** Jun-He Liu, Yan Yan, Abid Ali, Ming-Fu Yu, Qi-Jie Xu, Pei-Jian Shi, Lei Chen

**Affiliations:** 1 College of Biological and Food Engineering, Huanghuai University, Zhumadian, Henan 463000 P.R. China; 2 Landscape Research Institutes of Zhumadian, Zhumadian, Henan 463000 P.R. China; 3 Department of Entomology, University of Agriculture, Faisalabad, 38040 Pakistan; 4 College of Chemistry and Pharmaceutical Engineering, Huanghuai University, Zhumadian, Henan 463000 P.R. China; 5 Collaborative Innovation Centre of Sustainable Forestry in Southern China of Jiangsu Province, Nanjing Forestry University, 159 Longpan Road, Xuanwu, District Nanjing 210037 P.R. China; 6 Graduate School of Environmental Science, Hokkaido University, N19W8, Sapporo, 060–0819 Japan

## Abstract

Models that accurately estimate maximum crop biomass to obtain a reliable forecast of yield are useful in crop improvement programs and aiding establishment of government policies, including those addressing issues of food security. Here, we present a new sigmoidal growth model (NSG) and compare its performance with the beta sigmoidal growth model (BSG) for capturing the growth trajectories of eight crop species. Results indicated that both the NSG and the BSG fitted all the growth datasets well (*R*^2^ > 0.98). However, the NSG performed better than the BSG based on the calculated value of Akaike’s information criterion (AIC). The NSG provided a consistent estimate for when maximum biomass occurred; this suggests that the parameters of the BSG may have less biological importance as compared to those in the NSG. In summary, the new sigmoidal growth model is superior to the beta sigmoidal growth model, which can be applied to capture the growth trajectory of various plant species regardless of the initial biomass values at the beginning of a growth period. Findings of this study will be helpful to understand the growth trajectory of different plant species regardless of their initial biomass values at the beginning of a growth period.

## Introduction

The growth of plants is driven by numerous functions and involves numerous physiological and ecological processes^1,2^. The complete growth trajectory of a plant often resembles a sigmoidal curve^3,4^ that begins with a slow rate of increase, transitions to a log increase in growth rate and ends with a decrease in the rate of increase ending in zero growth when maximum biomass is reached. Although many growth models have been proposed for plants, such as the logistic, Richards and Gompertz growth models^1,5^, these models do not accurately predict the amount of maximum biomass present when the asymptotic line of the model trajectory, *w*_*max*_, is reached. To address this deficiency, the beta distribution function to describe the trajectory of plant growth rate was introduced^6,7^:

1 $$\frac{dw}{dt}={c}_{m}{[(\frac{{t}_{e}-t}{{t}_{e}-{t}_{m}}){(\frac{t-{t}_{b}}{tm-{t}_{b}})}^{\frac{{t}_{m}-{t}_{b}}{{t}_{e}-{t}_{b}}}]}^{{\boldsymbol{\delta }}}$$

where *w* is the biomass at time *t*, *δ* is a constant, *c*_*m*_ is the maximum growth rate, reached at time *t*_*m*_; *t*_*b*_ and *t*_*e*_ represent times of starting and ending growth, respectively. When *t*_*b*_ and *δ* are set as 0 and 1, respectively, the beta function can be simplified as follows:

2 $$\frac{dw}{dt}={c}_{m}(\frac{{t}_{e}-t}{{t}_{e}-{t}_{m}}){(\frac{t}{{t}_{m}})}^{\frac{{t}_{m}}{{t}_{e}-{t}_{m}}}$$

Then, the beta sigmoidal growth (hereafter read as BSG) model was derived by integrating the beta distribution function in Eq. (2):

3 $$w={w}_{max}(1+\frac{{t}_{e}-t}{{t}_{e}-{t}_{m}}){(\frac{t}{{t}_{m}})}^{\frac{{t}_{m}}{{t}_{e}-{t}_{m}}}(0\le {t}_{m} < {t}_{e})$$

where *t*_*e*_ is the time when biomass reaches the value of *w*_*max*_. In particular, the biomass *w* equals zero when *t* = 0 in Eq. (3). This assumption matches the growth trajectory of many plants; however, exceptions do occur. To address this issue, only assumed *δ* = 1 in Eq. (1) and then derived a more flexible beta sigmoidal growth model (BSG) in Eq. (4) to capture the growth trajectory of various plant species regardless of the initial biomass values^8^:

4 $$w={c}_{m}.(t-{t}_{b}).\frac{2{t}_{e}-{t}_{m}-t}{2{t}_{e}-{t}_{m}-{t}_{b}}{(\frac{t-{t}_{b}}{{t}_{m}-{t}_{b}})}^{\frac{{t}_{m}-{t}_{b}}{{t}_{e}-{t}_{m}}}$$

Although the BSG is slightly different from the leaf-growth model^9^, the BSG can produce similar growth curves to those obtained by the leaf-growth model^8^. In addition, the performance of the BSG is superior to other traditional growth models (e.g., Gompertz and von Bertanffy growth models) for its flexibility, outcompeting the exponential and logistic^8^. In this study, we developed and tested a new sigmoidal growth (NSG) model by comparing it with the beta sigmoidal growth (BSG) model. We evaluated both the models using observed growth datasets of eight crop species to estimate the timing and the maximum biomass when growth ceases.

## Results

**Figure 1:**
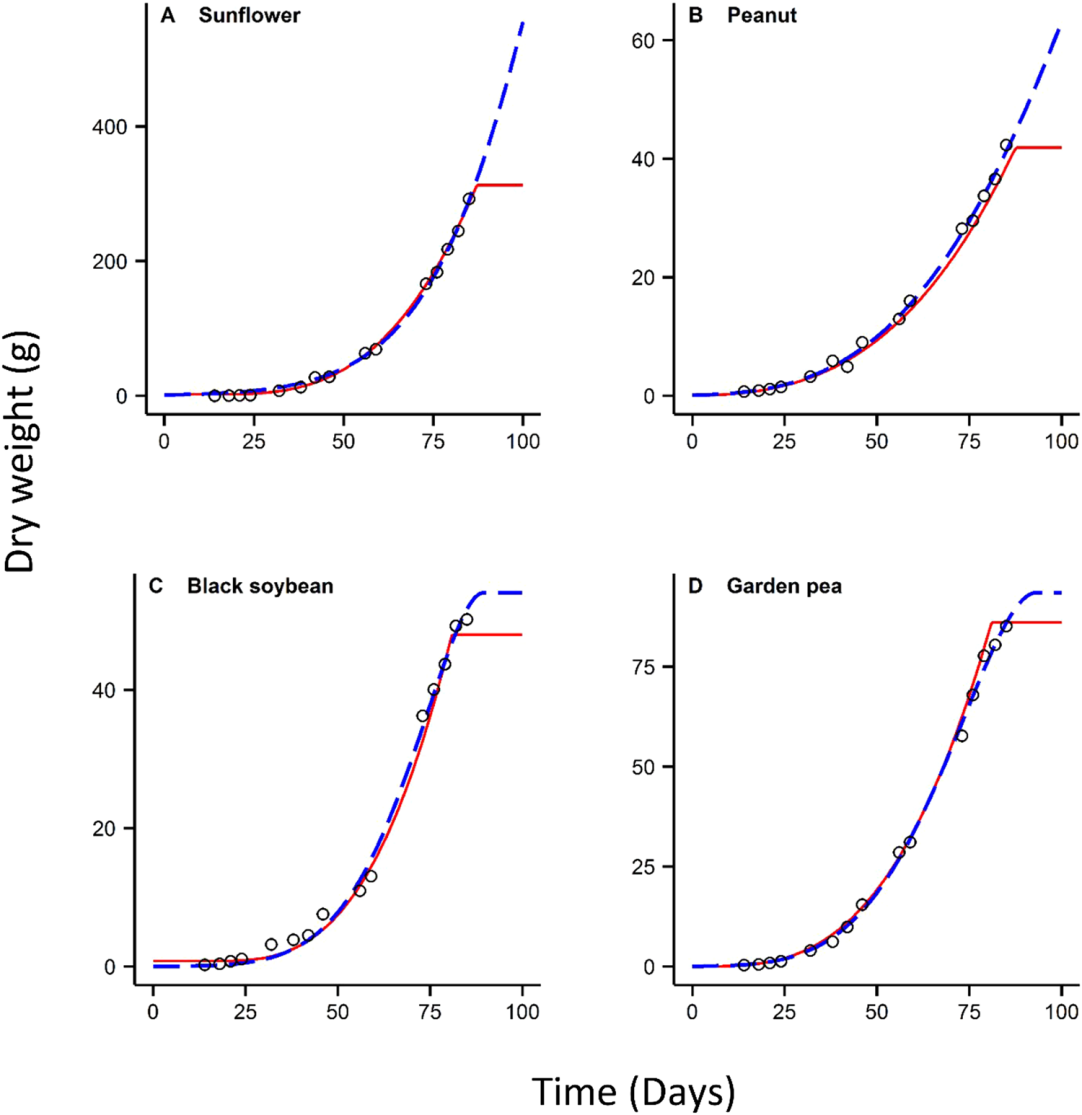
Comparison of fitted growth curves of the new growth model and the beta growth model for the growth datasets of sunflower, peanut, black soybean and garden pea. Circles represent the actual biomass observed. Red lines and black dashed lines represent the fitted curves of the new sigmoidal growth model and the beta sigmoidal growth model, respectively.

**Figure 2:**
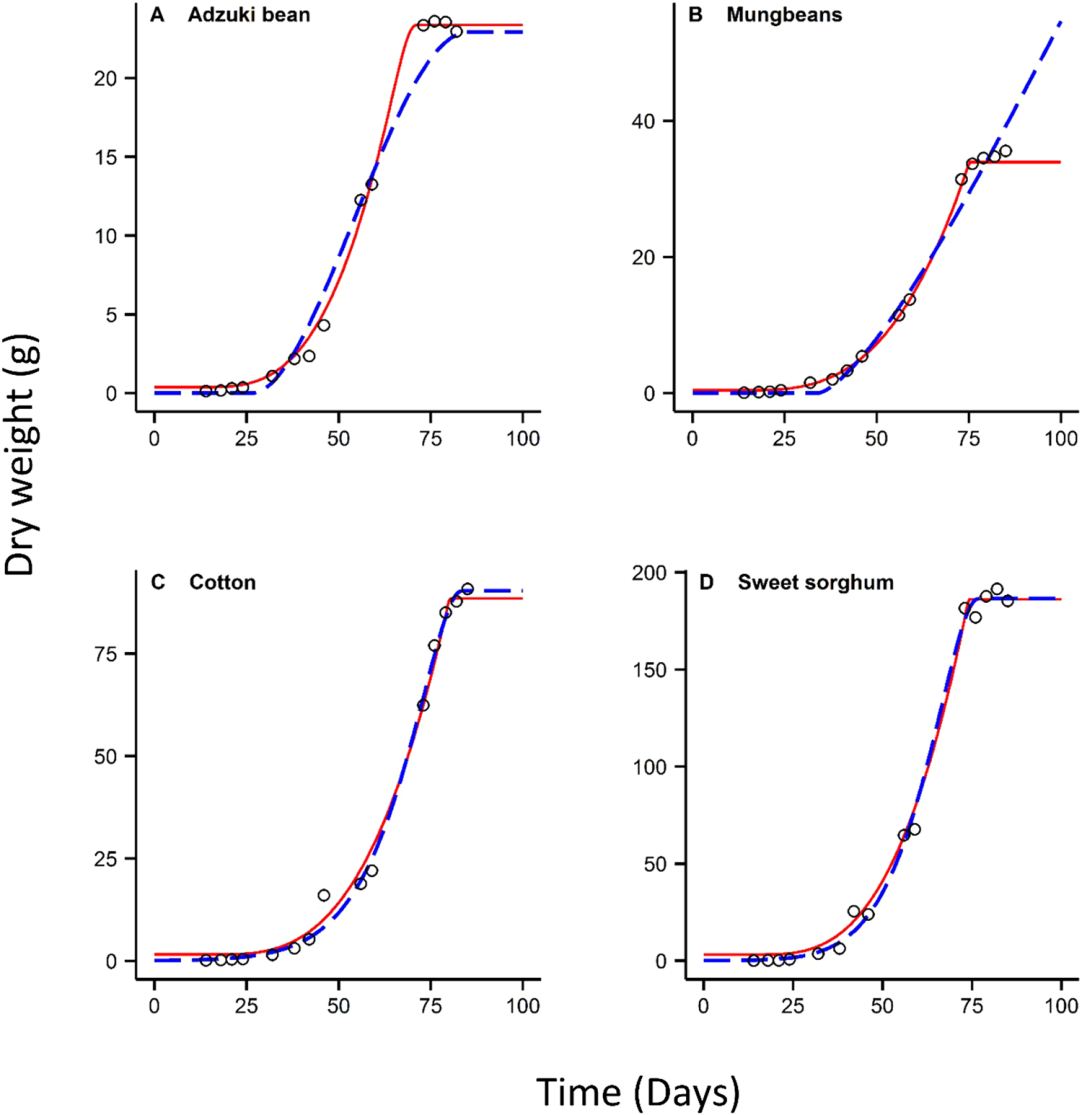
Comparison of fitted growth curves of the new growth model and the beta growth model in Eq. (4) for the growth datasets of adzuki bean, mung beans, cotton and sweet sorghum. Circles represent the actual biomass observed. Red lines and black dashed lines represent the fitted curves of the new sigmoidal growth model and the beta sigmoidal growth model, respectively.

Results showed the new sigmoidal growth (NSG) and the beta sigmoidal growth (BSG) provided good fits for all eight crop species well (*R*^2^ > 0.98) (see Table 1). The fitted curves of the NSG and BSG were generally close to each other before reaching the maximum biomass (Figs 1 and 2). The NSG performed better than the BSG based on the calculated values of Akaike’s information criterion (AIC) for the growth datasets (Table 1). The negative estimate of *t*_*b*_ from the NSG was observed only for peanut *Arachis hypogaea* L., whereas the estimates of *t*_*b*_ from the BSG were negative for six species. The estimated values of *t*_*e*_ from the BSG were higher than the NSG for all eight-crop species (Table 2). Similarly, the calculated values of maximum biomass (*w*_max_) of the BSG were all higher than those of the NSG except for the growth of Adzuki bean *Vigna angularis* (Wild.) (Table 3). For example, the estimates of *t*_*e*_ from the BSG for Mung bean reached 311 days, which was more than four times of that from the NSG. Because the observed value of biomass remained steady when the Mung bean reached 80 days of age (Fig. 2), the observed values of *t*_*e*_ and *w*_max_ were around 80 days and 40 g that compare well with the NSG model but not the BSG model that seriously overestimated the time/yield end points (311 days and 202 g, respectively). These results suggest that the estimates of *t*_*e*_ and calculated value of *w*_max_ from the BSG for is not reliable for some cases.

**Table 1:** Akaike’s information criterion (AIC) and R square values of the two growth models for the datasets of crop species.

Number of Species	Latin name	English name	NSG		BSG	
			*R* ^2^	AIC	*R* ^2^	AIC
1	*Helianthus annuus* L.	Sunflower	0.998	50.09	0.998	53.4
2	*Arachis hypogaea* L.	Peanut	0.997	−1.61	0.997	−1.14
3	*Glycine max* (L.) Merr.	Black soybean	0.996	15.05	0.995	15.74
4	*Pisum sativum* L.	Garden pea	0.998	17.21	0.998	18.35
5	*Vigna angularis* (Willd.) Ohwi et Ohashi	Adzuki bean	0.997	−9.13	0.982	16.66
6	*Vigna radiata* (L.) R. Wilczek	Mungbeans	0.999	−17.81	0.985	26.07
7	*Gossypium* spp.	Cotton	0.994	38.2	0.996	34.08
8	*Sorghum bicolor* (L.) Moench	Sweet sorghum	0.996	58.16	0.996	56.62

NSG and BSG represent the new sigmoidal growth model and the beta sigmoidal growth model in Eq. (4), respectively.

**Table 2:** Estimated parameters of the new and beta sigmoidal growth models for the growth datasets of crop species.

Species	NSG			BSG				
	*c*	*k*	*t*_*b*_	*t*_*e*_	*c*_*m*_	*t*_*m*_	*t*_*b*_	*t*_*e*_
Sunflower	−0.049	3.872	14.191	87.525	21.810	105.101	−251.198	121.770
Peanut	−0.012	2.788	−8.039	88.088	1.919	127.206	−18.244	176.192
Black soybean	−0.023	7.015	16.458	81.110	1.599	74.514	−24.915	89.598
Garden pea	−0.023	9.727	2.284	81.174	2.267	73.590	−17.770	93.217
Adzuki bean	−0.021	0.453	13.904	71.433	0.579	54.271	26.594	85.154
Mung bean	−0.021	22.886	14.052	75.209	1.062	116.547	34.286	311.459
Cotton	−0.032	26.624	16.679	80.116	3.465	73.278	−252.860	83.296
Sweet sorghum	−0.052	5.946	15.264	74.294	7.628	66.625	−54.308	76.695

NSG and BSG represent the new sigmoidal growth model and the beta sigmoidal growth model in Eq. (4), respectively.

**Table 3:** Estimated value of maximum biomass of the new and beta sigmoidal growth models for the growth datasets of crop species.

Species	NSG	BSG
	Maximum biomass (g)	Maximum biomass (g)
Sunflower	310.69	924.76
Peanut	41.90	177.78
Black soybean	47.97	54.09
Garden pea	86.07	93.56
Adzuki bean	23.36	22.92
Mung bean	33.92	202.84
Cotton	88.49	90.26
Sweet sorghum	186.24	186.40

NSG and BSG represent the new sigmoidal growth model and the beta sigmoidal growth model in Eq. (4), respectively.

## Discussion

Results indicated that both the NSG and the BSG can fit the growth datasets of crop species well, and NSG performed better than the BSG based on the calculated values of AIC (Table 1). The parameter *t*_*b*_ in the NSG is equivalent to the parameter *T*_1_ in Eq. (5), the lower threshold of temperature for the growth rate of bacteria. Ratkowsky *et al*.^10^ have pointed out that the parameter *T*_1_ in degrees Kelvin is a conceptual temperature representing an intrinsic feature of the organism^10^. In the study of Ratkowsky *et al*.^10^, a total of 13 estimates of *T*_1_ from the Eq. (5) are negative with lower than 273 K among all 16 bacterial cultures^10^. Accordingly, the estimates of *t*_*b*_ from the NSG can also assume negative values. As a result, estimates of *t*_*b*_ from the NSG were negative for the growth of peanut.

Although it observed that *t*_*b*_ could represent the beginning of the growth period at which the growth rate is set to zero^7^. However, our results showed that the estimates of *t*_*b*_ from the BSG were negative except for the Adzuki bean and Mung bean. Furthermore, the estimates of *t*_*e*_ from the BSG are not reliable for some cases (Table 2). For the BSG, the parameters of *t*_*b*_ and *t*_*e*_ represent the replacement of the lower and upper bounds, respectively, in the beta distribution function with four parameters. Consequently, the biological functions used for the parameters of *t*_*b*_ and *t*_*e*_ are artificially provided in the BSG model. The BSG fits the growth datasets of the eight crop species well (*R*^2^ > 0.98) (Table 1) through the flexibility of the beta distribution function, however, the parameters used to derive the BSG are not biologically accurate. Consequently, the estimates of *t*_*b*_ and *t*_*e*_ from the BSG are not biologically relevant in some cases, which is confirmed by the overestimated *w*_max_ compared to the observed values. On the contrary, the observed values of *t*_*e*_ and *w*_max_ compared well with the estimates of NSG model. Therefore, we concluded the NSG is better than the BSG and other traditional sigmoidal growth models, which can be applied to capture the growth trajectory of various plant species regardless of the initial biomass values at the beginning of the growth period.

## Methods

### Model derivation

Ambient temperature is the principal variable that determines the developmental growth rate of poikilotherms, including plants, which occurs in a range between the lower and upper developmental temperature threshold determined for each species. A nonlinear model was proposed to describe the effect of temperature on the growth rate of bacteria^10^:

5 $$\sqrt{r}=c(T-{T}_{1})(1-{e}^{k(T-{T}_{2})})$$

where *r* is the growth rate, *c* and *K* are constants, *T*_1_ and *T*_2_ is the minimum and maximum temperature for growth, respectively. After replacing the temperature with time, we derived a new sigmoidal growth model (NSG) by integrating Eq. (5) as follows:

6 $$w=\frac{1}{3}g(t)+\frac{{c}^{2}(\phi (t)+\theta (t)\eta (t)-16\eta (t)}{4{k}^{3}}$$

With $\eta (t)={e}^{k(T-{T}_{e})}$, $g(t)={c}^{2}{t}^{3}-3{c}^{2}{t}^{2}{t}_{b}+3{c}^{2}{t}_{b}^{2}t$, $\phi (t)=-\,8{k}^{2}{t}^{2}+16{k}^{2}t{t}_{b}-8{k}^{2}{t}_{b}^{2}+16kt-16k{t}_{b}$ and $\theta (t)=(2{k}^{2}{t}^{2}-4{k}^{2}t{t}_{b}+2{k}^{2}{t}_{b}^{2}-2kt+2k{t}_{b}+1){e}^{(kt-{t}_{e})}$ where *w* is the biomass at time *t*; *c* and *k* are constants; *t*_b_ and *t*_e_ represent the starting and ending time of growth.

### Parameter estimation

The parameters of the BSG and the NSG were estimated using the Differential Evolution by optimizing the problem and the Nelder-Mead algorithms by non-linear optimization problems to minimize the sum of squared errors between observed and predicted values, implemented in the R package DEoptim and the optim function of R^11^, respectively. We used the coefficient of determination *R*^2^ to evaluate the goodness of fit of models. The Akaike’s information criterion (AIC)^12^ was adopted to evaluate the goodness of fit and compare the performance of the NSG and BSG. A total of eight crop species^13^ (sunflower, peanut, black soybean, garden pea, adzuki bean, mung bean, cotton and sweet sorghum) were used for testing the two sigmoidal growth models. Both the NSG and the BSG assumed that the maximum biomass was reached at time *t*_*e*_, when the growth rate decreases to zero. Although the NSG and the BSG do not have the parameter of *w*_*max*_ like other sigmoidal growth models, the maximum biomass can be calculated after obtaining the estimates of *t*_*e*_. Therefore, the calculated values of maximum biomass of the NSG and the BSG were also compared in this study. However, based on these findings, there is dire need to incorporate the future work including spatial effects^14^ and different vegetation regimes^15^.
